# Changes in Feeding Behavior and Feeding Stress Among Mothers of Preschoolers Before and During the Novel Coronavirus Pandemic

**DOI:** 10.3389/fnut.2022.828557

**Published:** 2022-04-25

**Authors:** Rana H. Mosli, Alaa Barahim, Lina A. Zahed, Sara A. Ishaq, Fatimah M. Al-Eryani, Warda A. Alharbi, Hebah A. Kutbi, Haneen Saleemani

**Affiliations:** Clinical Nutrition Department, Faculty of Applied Medical Sciences, King Abdulaziz University, Jeddah, Saudi Arabia

**Keywords:** maternal feeding behavior, homeschooling, COVID-19 pandemic, feeding stress, lockdown 2020, preschool

## Abstract

**Objective:**

To examine changes in maternal feeding behaviors (MFB) and maternal feeding stress (MFS) among mothers of preschoolers in Saudi Arabia before and during the novel coronavirus pandemic.

**Methods:**

This is a prospective cohort study including 64 mothers of preschoolers who were drawn from a sample of a 2019 study. Study questionnaire was completed during November–December 2020. MFB were assessed using the Child Feeding Questionnaire-Arabic (CFQ-A) and MFS was assessed using the MFS-mealtimes index and the MFS-maternal resentment/difficult child index. Paired samples *t*-tests were used to examine changes in MFB and MFS.

**Results:**

Mothers have reported an increase in perceived responsibility (*M* = 4.09, SD = 0.87 vs. *M* = 4.33, SD = 0.59, *P*-value < 0.05) and monitoring (*M* = 4.23, SD = 0.73 vs. *M* = 4.48, SD = 0.66, *P*-value < 0.05) during the pandemic period compared to the period prior to the pandemic. Mothers have reported a decrease in use of food as a reward (M = 4.11, SD = 0.87 vs. *M* = 2.30, SD = 0.88, *P*-value **<** 0.001) and concern about child's diet (*M* = 3.34, SD = 1.12 vs. *M* = 2.55, SD = 1.04, *P*-value **<**0.001). There was an increase in MFS-maternal resentment/difficult child (*M* = 2.47, SD = 0.68 vs. *M* = 2.71, SD = 0.72, *P*-value < 0.01).

**Conclusions:**

Findings can help inform future research aiming to measure the long-term effects of the pandemic on child outcomes.

## Introduction

Mothers are usually primarily responsible for managing meals and the process of child feeding. Maternal feeding behaviors have been found to play an important role in shaping child eating behaviors and dietary patterns (1). Maternal concern about child's weight has been previously linked to using restrictive feeding practices, such as constraining child's access to specific foods. Nonetheless, restrictive feeding practices have been primarily observed among mothers who perceived their daughters to be overweight, while the use of pressure to eat has been observed among mothers who perceived their daughters to have a poor appetite or to be underweight (2, 3). Controlling/restrictive feeding behaviors are considered maladaptive behaviors, that have been previously associated with negative child outcomes, including elevated body mass index (BMI) and obesity proneness (2, 4, 5). However, responsive and supportive feeding behaviors, such as monitoring and perceived responsibility, are considered adaptive feeding behaviors (5, 6).

Variations in maternal feeding behaviors and strategies may exist among cultures. Arab/Middle Eastern mothers residing in Saudi Arabia (SA) have shown greater monitoring, perceived responsibility, and restriction in feeding compared to mothers from other Asian and Western countries (6). Qualitative data obtained through one-on-one interviews showed that Saudi mothers tend to invest an ample amount of emotional and physical energy in order to get their preschoolers to eat; They have reported cooking special meals for their child, decorating their child's plate in an attempt to make food more appealing, and using rewards to encourage the child to eat (7). These mothers also reported viewing feeding as a highly stressful and daunting process, and they have therefore been considered to have “high feeding stress” (7). A later study revealed that higher maternal feeding stress may mediate the association of child food fussiness and concern about child's diet with pressuring the child to eat (8).

Coronaviruses are a large number of viruses that cause cold and flu-like symptoms and may lead to potentially fatal severe illness that primarily affecting the respiratory system (9, 10). Following the high incidence of coronavirus infections, governments have announced lockdowns to mitigate the spread of the disease (10). The first case of COVID-19 in SA was reported on March 2nd, 2020 (11). On March 25th, 2020, the government announced the implementation of stringent lockdown policies and precautionary measures which significantly helped control the spread of the disease; City-wide curfews were instigated and in-person classes in schools and universities were suspended; Virtual learning using online platforms was adopted (12). School and daycare closures due to the novel coronavirus pandemic during the years 2020 and 2021 have led to increased burden and stress especially on working mothers who need to meet work, homeschooling and childcare responsibilities (13–16). Higher maternal stress in general may contribute to a maladaptive feeding environment, and more controlling feeding behaviors, including restriction and pressure to eat (17, 18).

Although many studies in the available literature have focused on maternal feeding behaviors (MFB) and maternal feeding stress (MFS) (7, 16, 17), no studies have targeted Saudi mothers' feeding behaviors and feeding stress during the novel coronavirus pandemic. A study conducted among mothers of school-age children in the United States (US) found that using food as a reward during the pandemic was related to the number of COVID-19-related life changes (13). Others found that parental monitoring and concern about child's weight, (19) in addition to restriction and pressure to eat (17), have increased among US families during the pandemic compared to the period before the pandemic (data collected retrospectively). Additionally, a study in France which also collected data retrospectively to assess changes in feeding practices during the pandemic found that parents changed their practices to become more permissive (20). To our knowledge, prospective data comparing maternal feeding behaviors (MFB) and maternal feeding stress (MBS) before and during the pandemic are scarce. Although families in SA may have experienced unique lockdown policies and precautionary measures, no previous study has examined changes in MFB and MFS among Saudi mothers before and during the pandemic. The overall aim of this study was to examine changes in MFB and MFS among Saudi mothers before and during the pandemic by following up with a cohort of mothers who participated in a study assessing MFB and MFS shortly prior to the pandemic, and re-assessing these constructs after the onset of the pandemic and nation-wide lockdowns. Findings from the present study can help inform counselling strategies and interventions aiming to improve MFB and MFS during the novel coronavirus pandemic (21), which can help promote a healthier mealtime environment and improve child eating behaviors and dietary intake (6). Results may give insights regarding the effects of the pandemic and inform future programs for children who have experienced the pandemic during early childhood years (17).

## Methods

### Study Design and Participants

This is a prospective study examining changes in MFB and MFS before and during the pandemic. Study participants were drawn from a previous sample of mothers who participated in a 2019 study examining the association between MFS and BMI z-score among preschoolers in SA (*n* = 100) (8). The 2019 study was completed prior to the onset of the pandemic. Of the 100 original participants, 36 had missing contact information or declined to participate. Thus, our final sample for the current analysis was 64 participants. Inclusion criteria were: Mother of a 4–6-year-old child, child is primarily living with the mother; child is not suffering from any serious health conditions or severe food allergies and is a permanent resident of SA; mother is fluent in Arabic. Verbal consent was obtained from mothers prior to data collection. Ethical approval from the Research and Ethics Committee at the Faculty of Applied Medical Sciences at King Abdulaziz University was obtained.

### Study Protocol

The study questionnaire was administered during the months of November and December 2020 by trained researchers. During an online voice call, researchers read questions and response questions aloud while displaying the questions on the screen. Participants were informed at the beginning of the interview that the current study was an extension of the previous study in which they had participated in during the year 2019, prior to the pandemic (8). The questionnaire was a replica of the previous questionnaire completed in 2019 and included the same items which assessed MFB and MFS.

### Measures

The Child Feeding Questionnaire-Arabic (CFQ-A) was used to assess MFB (2, 6). The questionnaire was translated into Arabic and validated among 209 Saudi mothers of preschoolers (6). It consists of 38 items used to derive the seven original CFQ factors (perceived responsibility, perceived parent weight, perceived child weight, concern about child weight, restriction, pressure to eat, and monitoring), in addition to two factors that were added to the modified CFQ-A: Use of food as a reward and concern about child's diet. The response options were 5-point Likert response scales ranging from 1 = “never” to 5 = “always”. Higher factor scores indicate a greater level of the given behavior. Internal reliability calculated as Cronbach's Alpha for each of the CFQ-A factors ranged from (α = 0.60 to α = 0.83); Cronbach's alpha values of at least 0.60 are considered “good”, while alphas of at least 0.70 are considered “favorable” (22).

MFS was assessed using the MFS-mealtimes index and the MFS-maternal resentment/difficult child index (8). The MFS-mealtimes index consists of 11 items adapted from the Depression Anxiety stress scale (DASS-21) (response options ranging from 0 = Did not apply to me at all to 3 = Applied to me very much, or most of the time) (23). The DASS-21 is a self-administered questionnaire designed to assess the degree of depression, anxiety, and stress. The DASS-21 was previously translated into Arabic and validated in a sample of Australian immigrant (24). MFS-mealtimes score was calculated as a mean of contributing items. Examples of MFS-mealtimes items include: “I tended to over-react to situations during mealtimes/feeding times with my child” and “I felt that I was using a lot of nervous energy during mealtimes/ feeding times with my child”. MFS-maternal resentment/difficult child index consists of 9 items adapted from the Parenting Stress Index (PSI)-short form (8, 25) (response options ranging from 0 = strongly disagree to 4 = strongly agree). MFS- maternal resentment/difficult child score was calculated as a mean of contributing items. Examples of MFS- maternal resentment/difficult child items include: “I didn't enjoy myself during mealtimes outside the home (e.g., in restaurants or gatherings) due to difficulty in feeding my child” and “My child was more difficult when it comes to feeding compared to other children.” Higher scores indicate a greater level of MFS. Internal reliability for MFS-mealtimes and MFS-maternal resentment/difficult child was calculated as α = 0.83 and α = 0.82, respectively.

Mothers reported data regarding sociodemographic characteristics, including the child's date of birth, sex and nationality (Saudi vs. not), the mother's date of birth, nationality (Saudi vs. not), educational level (≥ college degree vs. < college degree), and total monthly income ( ≤ 10,000 SR vs. >10,000 SR) (10,000 SR is equivalent to 2,666 USD and is considered a threshold for poverty in SA) (26). Child and maternal age were calculated based on dates of birth and dates of interviews.

### Statistical Analysis

The study sample was described using descriptive statistics; Means (M) and standard deviations (SD) were calculated for continuous variables and counts and percentages were calculated for categorical variables. Paired samples *t*-tests were used to examine changes in MFB and MFS before and during the novel coronavirus pandemic. Additional variables for “differences in MFB” and “differences in MFS” were created by subtracting scores for MFB and MFS prior to the pandemic from MFB and MFS scores during the pandemic; Pearson correlations were used to evaluate intercorrelations among differences in MFB and MFS. IBM SPSS Statistics (version 27.0; Armonk, NY, USA) was used to conduct the analyses. Significance level was set at ∞ <0.05.

## Results

### Sample Characteristics

As shown in Table 1, mean child age was 5.39 years (SD = 0.63), and 60.9% of children were female. The majority of children and their mothers were Saudi (75 and 70.3% respectively). Mean maternal age was 34.2 years (SD = 6.30), and the majority of mothers (75%) had a college degree or higher. In addition, a large proportion of mothers (70.3%) reported that their families had a total monthly income of 10,000 SR or less.

**Table 1:** Sample characteristics (*n* = 64)^*^.

**Variable**
Child age, M (SD)	5.39 (0.63)
Child sex, *N* (%)
Male	25.0 (39.1)
Female	39.0 (60.9)
Child nationality, *N* (%)
Saudi	48.0 (75.0)
Non-Saudi	16.0 (25.0)
Maternal age, *M* (SD)	34.2 (6.30)
Maternal nationality, *N* (%)
Saudi	45.0 (70.3)
Non-Saudi	19.0 (29.7)
Maternal education, *N* (%)
< College degree	16.0 (25.0)
≥College degree	48.0 (75.0)
Total monthly income, *N* (%)
≤ 10,000 SR	45.0 (70.3)
>10,000 SR	19.0 (29.7)

* *Table showing means (M) and standard deviations (SD) or counts (n) and percentages (%)*.

### Changes in MFB and MFS Before and During the Novel Coronavirus Pandemic

Table 2 displays the changes in MFB and MFS before and during the novel coronavirus pandemic. Significant changes were observed in four out of the nine CFQ-A feeding behavior scales. Mothers have reported an increase in perceived responsibility (*M* = 4.09, SD = 0.87 vs. M = 4.33, SD = 0.59, *P*-value < 0.05) and monitoring (*M* = 4.23, SD = 0.73 vs. *M* = 4.48, SD = 0.66, *P*-value < 0.05) during the pandemic period compared to the period prior to the pandemic. On the contrary, mothers have reported a decrease in use of food as a reward (*M* = 4.11, SD = 0.87 vs. *M* = 2.30, SD = 0.88, *P*-value **< ** 0.001) and concern about child's diet (*M* = 3.34, SD = 1.12 vs. *M* = 2.55, SD = 1.04, *P*-value **< ** 0.001) during the pandemic period compared to the period prior to the pandemic.

**Table 2:** Comparison of maternal feeding behaviors and feeding stress before and during the novel coronavirus pandemic (*n* = 64)^*^.

	**Prior to COVID-19 pandemic *M* (SD)**	**During COVID-19 pandemic *M* (SD)**	***t***	***P*-value**
Perceived responsibility	4.09 (0.87)	4.33 (0.59)	−2.15	0.03
Perceived maternal weight	3.06 (0.41)	3.12 (0.53)	−0.98	0.33
Perceived child weight	2.78 (0.42)	2.84 (0.52)	−0.87	0.39
Concern about child weight	2.80 (1.22)	2.54 (1.39)	1.57	0.12
Restriction	3.71 (0.55)	3.87 (0.72)	−1.49	0.14
Pressure to eat	3.88 (0.83)	3.81 (1.02)	0.74	0.46
Monitoring	4.23 (0.73)	4.48 (0.66)	−2.15	0.03
Use of food as a reward	4.11 (0.87)	2.30 (0.88)	12.00	<0.001
Concern about child's diet	3.34 (1.12)	2.55 (1.04)	5.28	<0.001
MFS-mealtimes	0.79 (0.58)	0.74 (0.56)	0.52	0.49
MFS-maternal resentment/difficult child	2.47 (0.68)	2.71 (0.72)	−2.98	0.004

* *Differences in means examined using paired samples t-tests*.

Mothers have reported an increase in MFS-maternal resentment/difficult child during the pandemic period compared to the period prior to the pandemic (*M* = 2.47, SD = 0.68 vs. *M* = 2.71, SD = 0.72, *P*-value < 0.01). However, no significant change was reported in MFS-mealtimes (*M* = 0.79, SD = 0.58 vs. *M* = 0.74, SD = 0.56, *P*-value > 0.05).

### Intercorrelations Among Differences in MFB and MFS

There were small to moderate positive correlations between difference in maternal perceived responsibility and difference in perceived maternal weight (*r* = 0.35, *P*-value < 0.01) monitoring (*r* = 0.32, *P*- value < 0.01), use of food as a reward (*r* = 0.51, *P*-value < 0.01) and MFS-maternal resentment/difficult child (*r* = 0.33, *P*-value < 0.01). A greater difference in perceived maternal weight was correlated with a smaller difference in perceived child weight (*r* = −0.30, *P*-value < 0.05). On the other hand, A greater difference in perceived maternal weight was correlated with greater differences in use of food as a reward (*r* = 0.32, *P*-value < 0.01), MFS-mealtimes (*r* = 0.30, *P*-value < 0.05), and MFS-maternal resentment/difficult child (*r* = 0.26, *P*-value < 0.05). Difference in concern about child's weight was positively correlated with difference in use of food as a reward (*r* = 0.26, *P*-value < 0.05) and concern about child's diet (*r* = 0.50, *P*-value < 0.01) (Table 3).

**Table 3:** Intercorrelations between differences in feeding behaviors and feeding stress (*n* = 64)^1^.

	**Difference in perceived responsibility**	**Difference in perceived maternal weight**	**Difference in perceived child weight**	**Difference in concern about child weight**	**Difference in restriction**	**Difference in pressure to eat**	**Difference in monitoring**	**Difference in use of food as a reward**	**Difference in concern about child's diet**	**Difference in MFS-mealtimes**	**Difference in MFS-maternal resentment/ difficult child**
Difference in perceived responsibility	—										
Difference in perceived maternal weight	0.35^**^	—									
Difference in perceived child weight	−0.22	−0.30^*^	—								
Difference in concern about child weight	0.18	0.17	−0.13	—							
Difference in restriction	−0.23	−0.08	−0.08	0.17	—						
Difference in pressure to eat	−0.02	−0.05	0.02	0.02	0.13	—					
Difference in monitoring	0.32^**^	0.08	−0.16	0.03	0.18	0.11	—				
Difference in use of food as a reward	0.51^**^	0.32^**^	−0.20	0.26^*^	0.11	−0.00	0.16	—			
Difference in concern about child's diet	0.22	0.17	0.00	0.50^**^	−0.08	−0.11	−0.15	0.17	—		
Difference in MFS-mealtimes	0.18	0.30^*^	−0.21	0.13	0.09	0.16	0.08	0.34^**^	0.17	—	
Difference in MFS-maternal resentment/difficult child	0.33^**^	0.26^*^	−0.27^*^	0.09	−0.02	0.14	0.31^*^	0.21	−0.15	−0.01	—

1 *Intercorrelation between variables examined using pearson correlation*.

* *P-value < 0.05*.

** *P-value < 0.01*.

Furthermore, a greater difference in monitoring was correlated with a greater difference in MFS-maternal resentment/difficult child (*r* = 0.31, *P*-value < 0.05), and a greater difference in use of food as a reward was corelated with a greater difference in MFS-mealtimes (*r* = 0.34, *P*-value < 0.01) (Table 3).

## Discussion

Our data showed significant changes in reported MFB and MFS among mothers of preschoolers before and during the novel coronavirus pandemic. Findings showed an increase in maternal perceived responsibility and monitoring and a decrease in use of food as a reward and concern about child's diet. Furthermore, we have detected an increase in feeding stress (maternal resentment/difficult child) during the pandemic period compared to the period prior to the pandemic.

School closures and remote work during the novel coronavirus pandemic has led to family members spending most of their time together in the vicinity of their homes (10). Mothers being around their children during every meal of the day in comparison to only one to two meals prior to the pandemic when schools were open, might have led to the observed increase in perceived responsibility and monitoring. Further, parents might be more focused on the quality of food that family members are consuming during the pandemic as some may believe that a specific/healthy diet can protect from the coronavirus and its complications. Previous studies in the United States have also reported an increase in monitoring during the pandemic, in addition to an increase in restriction and pressure to eat (17, 19). Our study distinctly showed an increase in perceived responsibility during the pandemic, which might be related to Arab/Middle Eastern mothers generally reporting higher perceived responsibility compared to mothers from other cultures (6). Furthermore, the reduction in use of food as a reward and concern about child's diet might be related to mothers managing all meals and snacks in the household which might increase their sense of awareness of types of foods that the child is consuming throughout the day. Indeed, previous research has raised concern regarding the quality of meals served in schools, with predominance of unhealthy food items that lack necessary nutrients including iron, calcium, and vitamin D (27). Nonetheless, higher perceived responsibility has been previously linked to lower concern about child's diet (6). Further studies are needed in order to establish cross-cultural distinctions in changes in maternal feeding behaviors in response to the novel coronavirus pandemic.

Although the observed increase in perceived responsibility and monitoring and decrease in use of food as a reward and concern about child's diet in the wake of the pandemic might be describe as an overall positive change, changes in maternal stress levels might be unfavorable. Our finding of increased feeding stress (maternal resentment/difficult child) during the pandemic period might be explained by the increased burden and sense of responsibility as mothers became in charge of all meals and snacks during quarantine. Furthermore, elevated maternal feeding stress may also reflect the effects of the overall psychological distress associated with the pandemic. In line with our findings, a recent study has found that although efforts for positive interactions during mealtimes have increased during the pandemic, this was associated with increased parental stress levels (28). In-depth qualitative data are needed to further understand how changes in parental stress levels are linked to changes in feeding practices during the pandemic.

Our assessment of intercorrelations among differences in MFB and MFS showed that mothers who perceived themselves to have greater responsibility in feeding during the pandemic reported greater change in their perceived weight, monitoring their child's eating and using food as a reward, as well as a greater change in MFS-maternal resentment/difficult child. Additionally, mothers who reported greater change in their perceived weight during the pandemic also reported greater change in using of food as a reward, MFS-mealtimes, and MFS-maternal resentment/difficult child. A greater change in concern about child's weight was associated with a greater change in using food as a reward and concern about child's diet. Although previous reports showed that maternal feeding stress might be positively associated with pressuring the child to eat (8), additional research is needed to identify predictors of change in maternal feeding behaviors and feeding stress levels during the pandemic. Potential factors affecting change in feeding behaviors and stress during the pandemic may include change in employment status and food security and income level, as well as the health status of family members and perceived threat and risks of the coronavirus infection. Furthermore, although our analysis did not include data pertaining to fathers' involvement in feeding, future work may assess paternal behaviors and characteristics as predictors of change in MFB and MFS during the pandemic.

Findings from the present study can help inform future research that aim to evaluate the long-term effects of the novel coronavirus pandemic on children's weight and health outcomes through changes in MFB and MFS. Our results may also help inform intervention studies aiming to improve features of the home environment amid the pandemic. Limitations of our study include the relatively small sample size and inability to objectively collect weight and height data due to the pandemic circumstances. In addition, findings from this study may not be generalizable to the entire Saudi population as data were drawn from a single city in SA. Although fathers' involvement in feeding and parenting during the pandemic might have influenced changes in MFB and MFS, data pertaining to fathers were not included in our study. Future larger studies with representative samples that link changes in MFB and MFS to objectively collected anthropometric data are needed. Evaluation of fathers' influence on MFB and MFS is warranted. Our strengths include our prospective study design which enabled us to conduct the first study examining changes in feeding behaviors and feeding stress among mothers of preschoolers in SA during the novel coronavirus pandemic.

## Conclusion

The novel coronavirus has affected daily life in economic, social, and educational aspects (29). This study has highlighted changes in MFB and MFS during the pandemic. Further investigation of the effects of homeschooling and remote work on familial behaviors and child outcomes is warranted in order to help improve long-term outcomes of children and adults from various cultures and backgrounds.

## Data Availability Statement

The raw data supporting the conclusions of this article will be made available by the authors, without undue reservation.

## Ethics Statement

The studies involving human participants were reviewed and approved by Research and Ethics Committee at the Faculty of Applied Medical Sciences at King Abdulaziz University. Written informed consent to participate in this study was provided by the participants.

## Author Contributions

RM, HS, AB, LZ, SI, FA-E, and WA designed the study and organized data collection. RM, HS, AB, SI, FA-E, WA and HK analyzed the data. All authors critically reviewed the manuscript, and approved the final version as submitted.

## Conflict of Interest

The authors declare that the research was conducted in the absence of any commercial or financial relationships that could be construed as a potential conflict of interest.

## Publisher's Note

All claims expressed in this article are solely those of the authors and do not necessarily represent those of their affiliated organizations, or those of the publisher, the editors and the reviewers. Any product that may be evaluated in this article, or claim that may be made by its manufacturer, is not guaranteed or endorsed by the publisher.
